# Alternative dosing of dual PI3K and MEK inhibition in cancer therapy

**DOI:** 10.1186/1471-2407-12-612

**Published:** 2012-12-21

**Authors:** Elina Jokinen, Niina Laurila, Jussi P Koivunen

**Affiliations:** 1 Department of Medical Oncology and Radiotherapy, Oulu University Hospital, Oulu PB22 90029 OYS, Finland

**Keywords:** Non-small cell lung cancer, PI3K inhibition, MEK inhibition

## Abstract

**Background:**

PI3K/AKT/mTOR and RAS/RAF/MEK/ERK pathways are thought to be the central transducers of oncogenic signals in solid malignancies, and there has been a lot of enthusiasm for developing inhibitors of these pathways for cancer therapy. Some preclinical models have suggested that combining inhibitors of both parallel pathways may be more efficacious, but it remains unknown whether dual inhibition with high enough concentrations of the drugs to achieve meaningful target inhibition is tolerable in a clinical setting. Furthermore, the predictive factors for dual inhibition are unknown.

**Methods:**

Non-small cell lung cancer (NSCLC) cell lines (n=12) with the most frequent oncogenic backgrounds (K-Ras mut n=3, EGFR mut n=3, ALK translocated n=3, and triple-negative n=3) were exposed to PI3K inhibitors (ZSTK474, PI-103) or MEK inhibitor (CI-1040) alone or in combination and analysed with an MTS growth/cytotoxicity assay and statistically by combination index analysis. The activity of the intracellular signaling pathways in response to the inhibitor treatments was analysed with a western blot using phospho-specific antibodies to AKT, ERK1/2, S6, and 4E-BPI. For the differential dosing schedule experiments, additional breast and colon cancer cell lines known to be sensitive to dual inhibition were included.

**Results:**

Two of the 12 NSCLC cell lines tested, H3122 (ALK translocated) and H1437 (triple-negative), showed increased cytotoxicity upon dual MEK and PI3K inhibition. Furthermore, MDA-MB231 (breast) and HCT116 (colon), showed increased cytotoxicity upon dual inhibition, as in previous studies. Activation of parallel pathways in the dual inhibition-sensitive lines was also noted in response to single inhibitor treatment. Otherwise, no significant differences in downstream intracellular pathway activity (S6 and 4E-BPI) were noted between PI3K alone and dual inhibition other than the increased cytotoxicity of the latter. In the alternative dosing schedules two out of the four dual inhibition-sensitive cell lines showed similar cytotoxicity to continuous PI3K and short (15min) MEK inhibition treatment.

**Conclusions:**

Therapy with a dual PI3K and MEK inhibitor combination is more efficient than either inhibitor alone in some NSCLC cell lines. Responses to dual inhibition were not associated with any specific oncogenic genotype and no other predictive factors for dual inhibition were noted. The maximal effect of the dual PI3K and MEK inhibition can be achieved with alternative dosing schedules which are potentially more tolerable clinically.

## Background

Constitutive activation of oncogenic pathways occurs in cancers with very high frequency, and this is thought to be a central factor behind the hallmarks of cancer phenotypes, such as cycle progression, inhibition of apoptosis and metabolic reprogramming. The PI3K-AKT and RAS-RAF-MEK-ERK pathways are thought to play a central role in transmitting these oncogenic signals. Frequent cancer-associated genetic alterations such as receptor mutations or amplifications, mutations in intermediate signal transducers such as *Ras*, *Raf* or *PI3KCA* and inactivation of certain tumor suppressors such as *PTEN* lead to constitutive activation of these pathways [1].

The high frequency of cancer-associated genetic alterations causing constitutive activation of PI3K-AKT and RAF-MEK-ERK and the addiction of cancer cells to their signals have led to enthusiasm for developing inhibitors of these pathways. In view of the central role of such pathways in transmitting upstream oncogenic signals, their inhibition could be an effective therapy for various cancer genotypes. Some cancer genotypes have been identified in preclinical studies as responders to specific inhibitors of the pathways. *HER2* amplified breast cancers have been shown to respond to PI3K inhibitors [2], while *BRaf* mutant melanomas [3] and triple-negative breast cancers are repressed by MEK inhibitors [4]. The effectiveness of single pathway inhibition could be suppressed by *de novo* dependence on multiple signaling pathways or feedback activation of other signaling pathways in response to the inhibition of a single pathway [2,5]. This has led to studies combining PI3K or AKT and MEK inhibitors. Dual inhibition has shown increased efficiency in various cancer genotypes in pre-clinical studies [2,4,6,7] and numerous early-phase clinical studies are in progress. Clinical studies have shown the simultaneous inhibition of multiple pathways to be in all probability more toxic than inhibition of a single pathway, and no optimal dose has been established.

PI3K-mTOR inhibitors may be divided into PI3K inhibitors (such as ZSTK474), dual PI3K–mTOR inhibitors (such as PI-103) and mTOR inhibitors (rapalogs). Rapalog mTOR inhibitors are known to induce IRS-1-mediated, upstream feedback activation of PI3K-AKT [8], which is thought to be important for the limited clinical efficiency of the therapy for most cancers, including NSCLC. PI3K and PI3K/mTOR inhibitors should lack such feedback activation and theoretically be more active. Numerous early phase clinical trials are currently testing both single PI3K and dual PI3K/mTOR inhibitors, but it is unknown whether either is more efficient, although it is likely that a drug which hits multiple targets will be more toxic in a clinical setting.

Current oncological therapies have modest disease modifying effects in cases of non-small cell lung cancer (NSCLC), even though some disease subgroups responsive to targeted therapy have been identified in recent years. These include *EGFR* mutant (10-30% of patients) [9,10] and *ALK* translocated (~5%) [11,12], in which patients are highly responsive to EGFR or ALK tyrosine kinase inhibitors (TKI) [13,14]. Furthermore, other major oncogenic disease subgroups include the *KRas* mutant (~25% of patients), which is thought to be undruggable with currently available pharmacological agents [15].

We set out here to investigate dual inhibition with PI3K and MEK in non-small cell lung cancer (NSCLC) cell lines of various genotypes. Dual inhibition is shown to be a more effective form of therapy in some cell lines. This study also addresses administration schedules for the inhibitors which may prove less toxic in a clinical setting.

## Methods

### Cell lines

The cell lines used here included NSCLC lines with a *K*-*Ras* mutation (A549, H358, H441), *EGFR* mutation (H1975, HCC827, PC-9), *ALK* translocation (DFCI032, H2228, and H3122) and the triple negative genotype (A431, H1437, H1581), a basal-like breast cancer line MDA-MB231 and HCT116, a *K*-*Ras* mutant colorectal cell line. The NSCLC cell lines were kind gifts from Dr. Pasi Jänne (Dana-Farber Cancer Institute, Boston, USA), and the breast and colorectal lines from Dr. Peppi Koivunen (Oulu University, Oulu, Finland). The cell lines were cultured in RPMI-1640 supplemented with 5 or 10% fetal bovine serum and 100 IU/ml penicillin and streptomycin. All the cell culture reagents were purchased from HyClone (Logan, UT).

### Inhibitors

The following inhibitors were used: CI-1040, PI-103, ZSTK474 (Alexis Biochemicals; Lausen, Switzerland), and TAE684 (a kind gift from Dr. Nathanael Gray, Dana-Farber Cancer Institute, Boston, USA). All the inhibitors were dissolved in DMSO to a final concentration of 10mM and stored at −20°C. The drug solutions for the experiments were prepared from a 10mM stock solution immediately before use. MEK inhibitor CI-1040 (PD-184352), a specific small-molecule drug that inhibits MEK1/MEK2, is thought to act as an allosteric inhibitor of MEK, since it is known not to compete with the binding of either ATP or protein substrates. CI-1040 blocks ERK phosphorylation and inhibits the growth of multiple human tumor cell lines and tumor growth in xenograft models. It has been shown that the inhibitory effect of CI-1040 on cell growth is rapidly reversed after it is removed from the growth medium [16]. ZSTK474 is a small-molecule PI3K inhibitor which has shown to be a potential antitumor agent against a human cancer xenograft *in vivo* with no toxicity to any critical organs [17]. It inhibits all four PI3K isoforms, most strongly PI3Kδ, by competing with the binding of ATP to the ATP-binding-pocket of the protein. In addition, the molecule is significantly specific to PI3K, since even when administered at high concentrations it only weakly inhibits the mTOR complex, which contains a conserved PI3K domain [18]. PI-103 is a pyridofuropyrimidine compound that selectively inhibits PI3Kα and mTOR signaling, prevents cell proliferation and invasion, causes G0-G1 cell cycle arrest and reduces tumor growth in glioma xenografts [19]. The inhibitor has also shown significant antitumor potency in NSCLC cell lines [20].

### Cytotoxicity/cell growth assay

Cells were plated onto 96-well plates with three to six parallel wells for each treatment, the experiments being replicated at least three times. The inhibitor treatments were started on the following day, and the plates were developed 72h later using an MTS reagent mix ([3-(4, 5-dimethylthiazol-2-yl)-5-(3-carboxymethoxyphenyl)-2-(4-sulfophenyl)-2H-tetrazolium, inner salt], Promega; Madison, WI) supplemented with phenazine methosulfate (Sigma-Aldrich; St. Louis, MO) according to the manufacturer’s guidelines. The absorbances were read on a plate reader (Athos Labtec Instruments; Salzburg, Austria) at a wavelength of 488nm. The data were displayed graphically using GraphPad Prism (GraphPad Software; La Jolla, CA), with the absorbance in the non-treated wells as the reference value (100%). The combination index (CI) was calculated using Calcusyn software (BIOSOFT, Cambridge, UK), and a 3.3:1 ratio of the PI3K inhibitors to the MEK inhibitor was used in the CI analysis. CI values at ED50 are presented.

### Western blot analysis

The cells were plated onto 6-well plates and treated with the drugs 24-48h later for 6 or 72 h, after which they were lysed in RIPA buffer (1% Igepal CA-630, 20 mM Tris–HCl pH 8.0, 137 mM NaCl, 10% glycerol, 2 mM EDTA, 1 mM sodium orthovanadate, 10 μg/mL Aprotinin, 10 μg/mL Leupeptin, and 10 μg/mL Pepstatin). Protein concentrations were measured using the Bio-Rad Protein Assay (Bio-Rad; Hercules, CA) and the concentrations in individual samples were equalized before adding 3x Laemmli buffer to a final concentration of 1x. Equal amounts of protein were run on 7.5% SDS-PAGE gels, transferred to PVDF membranes, probed with the antibodies and developed using the ECL chemiluminescence system (Millipore; Billerica, MA) for detection on radiographic films, which were scanned to an electronic format. All the antibodies used were from Cell Signaling Technologies (Danvers, MA): pAKT (S473), AKT, pERK (T202/Y204), ERK, pS6 (Thr389), S6, p4E-BP1 (Thr37/46), 4E-BP1, cleaved PARP. Anti-rabbit HRP conjugated antibody was used as a secondary antibody.

### Pathscan analysis

The PathScan analysis was carried out with the PathScan® RTK Signaling Antibody Array kit (Cell Signaling Technologies, Danvers, MA) according to the manufacturer’s guidelines. In brief, cells were plated on plates of diameter 6 cm and drugged the following day for 24 h. Whole cell lysates were collected, protein concentrations were determined using the Bio-Rad Protein Assay (Bio-Rad, Hercules, CA) and the protein concentrations were equalized. The lysates were applied to nitrocellulose membranes and incubated over night, washed, exposed to the secondary antibodies, developed with ECL and imaged with a Fujifilm LAS-3000 Luminescent Image analyzer and the ImageReader LAS-3000 program. The array target map can be found through the manufacturer’s homepage (http://www.cellsignal.com/products/7982.html).

## Results

### Dual inhibition of PI3K and MEK in cancer cell lines

**Figure 1:**
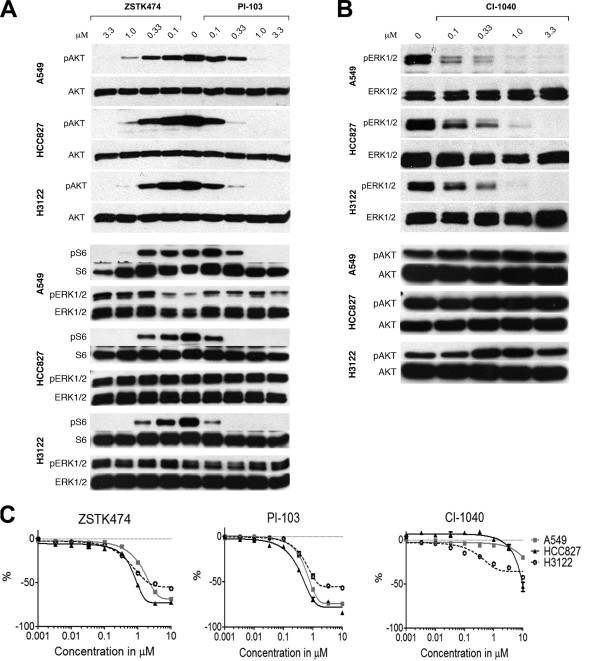
**PI3K and MEK inhibitors in NSCLC cell lines. **NSCLC cell lines A549, HCC827 and H3122 were exposed to increasing concentrations of the PI3K inhibitors ZSTK474 and PI-103 and the MEK inhibitor CI-1040. (**A**) Western blot analysis of phosphorylated AKT (pAKT), S6 (pS6), and ERK1/2 (pERK1/2) and their corresponding total proteins (AKT, S6, ERK1/2) in response to the PI3K inhibitors. (**B**) Western blot analysis of phosphorylated ERK1/2 (pERK1/2) and AKT (pAKT) and their corresponding total proteins (ERK1/2, AKT) in response to the MEK inhibitor. (**C**) MTS assay for cytotoxicity in response to the PI3K inhibitors or MEK inhibitor. The cells were exposed to the inhibitors for 6h in the Western blot experiments and 72 h in MTS assays. Error bars show SD.

The inhibitors used were ZSTK474 (PI3K inhibitor) and PI-103 (PI3K and mTOR inhibitor) and CI-1040 (MEK inhibitor). We first addressed the effects of these inhibitors alone in the NSCLC lines A549 (*K*-*Ras* mutant), HCC827 (*EGFR* mutant) and H3122 (*EML4*-*ALK* translocated), representing the three most frequent oncogenic genotypes of the disease, to establish concentration frames for the target inhibition. In the Western blots ZSTK474 at a 3.3μM concentration induced complete downregulation of pAKT, an immediate downstream target of PI3K, while PI-103 induced a similar inhibition at concentrations of 1 to 3.3 μM (Figure 1A). pS6 downregulation correlated highly with pAKT downregulation (Figure 1A). The MTS cytotoxicity assay showed a major reduction in the number of viable cells in all the cell lines with similar concentrations of both inhibitors, which were closely correlated with the concentrations inducing complete inhibition of pAKT in Western blot analysis (Figure 1A,C). CI-1040 induced complete inhibition of ERK1/2, an immediate downstream target of MEK, at a 1 μM concentration (Figure 1B). Only the H3122 line showed any marked reduction in cell viability in the MTS assays in response to increasing concentrations of the inhibitor, correlating with maximal target inhibition, while the other lines displayed minor changes in viability, except for the 10 μM treatment in HCC827, despite the achieving of complete inhibition of pERK1/2 in all the lines tested at 1 μM (Figure 1C).

**Figure 2:**
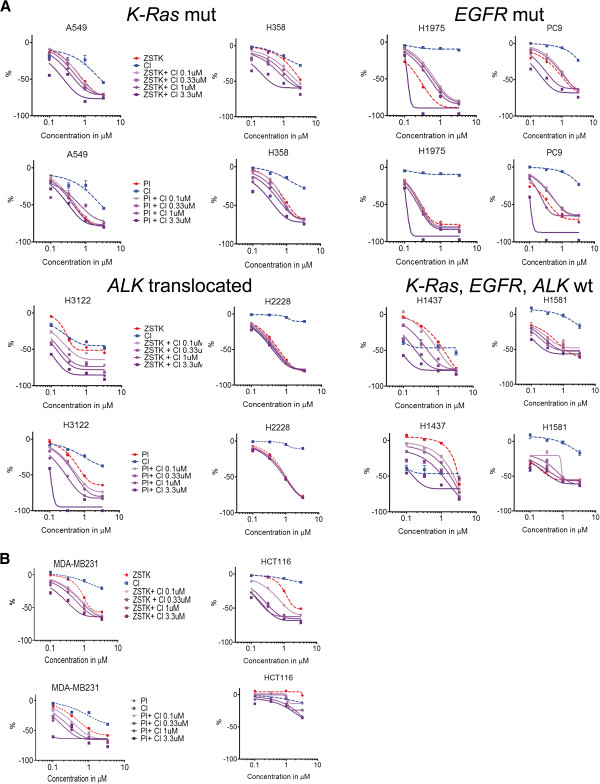
**PI3K and MEK inhibitors and their combinations in cancer cell lines. **The cells were exposed to specific treatments for 72 h and analyzed with the MTS cytotoxicity assay. The single inhibitor treatments are represented with dashed lines and their combinations with continuous lines. (**A**) NSCLC cell lines. The cell lines are placed in four groups based on their oncogenic genotype (*K*-*Ras *mutant, *EGFR *mutant, *ALK *translocated, and triple wild type). (**B**) Breast cancer line MDA-MB231 and colorectal cancer line HCT116, known from the previous literature to be sensitive to dual PI3K and MEK inhibition. Error bars show SD.

Dual inhibition of PI3K and MEK was tested in a panel of NSCLC lines (n=12) with the *K*-*Ras* (n=3), *EGFR* (n=3), *ALK* (n=3), or triple-negative (n=3) oncogenic genotypes. Analogously to the cell lines in the preliminary experiments, all the cell lines tested here showed a major reduction in cell growth in response to the PI3K inhibitors alone, with no significant differences between ZSTK474 or PI-103 (Figure 2A, eight of the twelve lines presented graphically). The MEK inhibitor CI-1040 elicited variable responses with the majority of cell lines, showing only minor inhibition of growth or none at all. When the cell lines were exposed to dual, concurrent inhibition of PI3K and MEK, two out of 12 tested cell lines, H3122 and H1437, showed marked additional cytotoxicity compared with treatment with a single agent (Figure 2A). The results were submitted to combination index (CI) analysis and average CI values were calculated based on combinations of ZSTK474 and PI-103. This analysis grouped the cell lines into three categories: antagonism (n=5, CI 1.10-3.3), nearly additive or slight synergy (n=5, CI 0.7-1.10), and synergy or strong synergy (n=2, CI <0.7) (Table 1). Visual assessment of the dual inhibition in MTS curves did not suggest any major antagonism of treatment in any of the lines tested, however, since the combination treatment curves in the cell lines with antagonistic CI values closely followed the single PI3K inhibitor treatment curves (Figure 2A). There was no correlation between the cancer genotypes in responsiveness to the dual inhibition, since an *ALK* translocated line (H3122) and a triple-negative negative line (H1437) showed synergistic responses to dual inhibition (Figure 2A, Table 1). The NSCLC lines showing synergistic responses to dual inhibition seemed to be more responsive to low concentrations (<1 μM) of the MEK inhibitor alone (Figure 2A). Analogously to the single inhibitor results, the lines sensitive to dual inhibition showed only a minor difference between the activities of the different PI3K inhibitors in combination with the MEK inhibitor.

**Table 1:** Combination index analysis

**Cell line**	**PI3K inhibitor**	**CI (ED50)**	**Average CI**	**Range**	**Significance**
H1437	ZSTK474	0,4	0,3	0,1-0,3	Strong synergism
H1437	PI-103	0,2			
H3122	ZSTK474	0,27	0,325	0,3-0,7	Synergism
H3122	PI-103	0,38			
MDA-MB231	ZSTK474	0,58	0,515	0,3-0,7	Synergism
MDA-MB231	PI-103	0,45			
HCT116	ZSTK474	0,4	0,635	0,3-0,7	Synergism
HCT116	PI-103	0,87			
DFCI032	ZSTK474	0,61	0,71	0,7-0,85	Moderate synergism
DFCI032	PI-103	0,81			
H358	ZSTK474	0,6	0,79	0,7-0,85	Moderate synergism
H358	PI-103	0,98			
H2228	ZSTK474	0,87	0,885	0,85-0,9	Slight synergism
H2228	PI-103	0,9			
A549	ZSTK474	0,9	0,93	0,90-1,10	Nearly additive
A549	PI-103	0,96			
H441	ZSTK474	1,14	1,07	0,90-1,10	Nearly additive
H441	PI-103	1			
A431	ZSTK474	1,17	1,19	1,10-1,20	Slight antagonism
A431	PI-103	1,21			
HCC827	ZSTK474	0,89	1,45	1,20-1,45	Moderate antagonism
HCC827	PI-103	2,01			
PC9	ZSTK474	1,37	1,65	1,20-1,45	Antagonism
PC9	PI-103	1,93			
H1518	ZSTK474	1,2	1,72	1,45-3,3	Antagonism
H1518	PI-103	2,24			
H1975	ZSTK474	2,3	1,745	1,45-3,3	Antagonism
H1975	PI-103	1,19			

**Figure 3:**
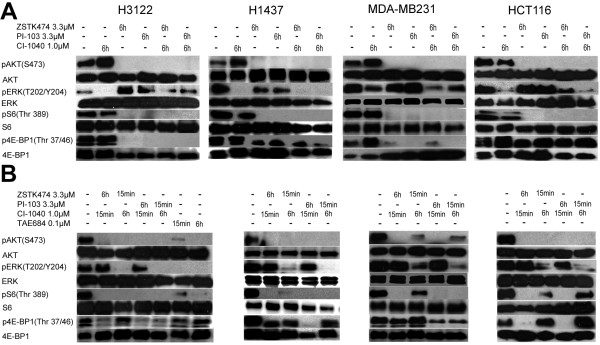
**Activity of the central cell signaling pathways in response to dual PI3K and MEK inhibition in dual inhibition**-**sensitive cancer cell lines. **(**A**) Western blot analysis for pAKT, pERK1/2, pS6 and p4E-BP1 and their corresponding total proteins in response to treatment with the PI3K inhibitors ZSTK474 and PI-103 and the MEK inhibitor CI-1040 singly or in combination for 6h. (**B**) Western blot analysis of the same proteins as in panel A when the inhibitor combinations were administered alternately for the times indicated (15 min or 6 h). The cells were exposed to dual inhibition for 15 min, the inhibitors were washed out and treatment was continued with a single inhibitor for an additional 6 h.

Based on a literature search [4,7], additional cell lines known to be responsive to dual PI3K and MEK inhibition were studied. MDA-MB231, a basal-like breast cancer line, and HCT116, a *KRas* mutant colorectal line, were exposed to single inhibitors or dual inhibition and analyzed with the MTS assay. As in the previous work, both the cell lines showed synergistic responses to dual inhibition (Figure 2B, Table 1). PI-103 was markedly less effective than ZSTK474 in the HCT116 cell line, while, like all the NSCLC cell lines, MDA-MB231 responded similarly to both PI3K inhibitors (Figure 2B, Table 1). Interestingly, we did not see any differences in target inhibition between ZSTK474 and PI-103 in the HCT116 line (Figure 3A), so that the mechanism of differential efficiency remains unknown.

**Figure 4:**
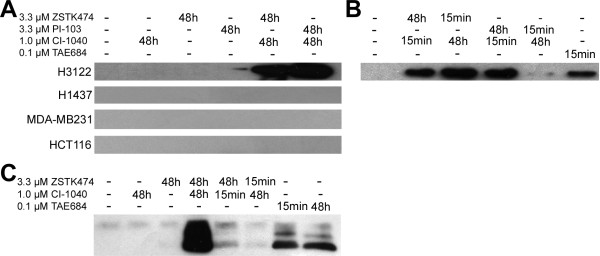
**Apoptotic response to dual PI3K and MEK inhibition in dual inhibition**-**sensitive cancer cell lines. **(**A**) Western blot analysis for cleaved PARP (cPARP) in the H3122, H1437, MDA-MB231 and HCT116 lines treated with the PI3K inhibitors ZSTK474 and PI-103, the MEK inhibitor CI-1040, or their combinations for 48 h. (**B**) Western blot analysis for cleaved PARP in the H3122 cell line treated for 15min with dual inhibition and with the single inhibitor indicated for an additional 48 h, or with the ALK inhibitor TAE684 for 15 min. (**C**) Western blot analysis for cleaved caspase-3 in the H3122 line with indicated treatments and exposure times.

The lines H3122, H1437, MDA-MB231, and HCT116, which were sensitive to dual inhibition, were further analyzed with Western blot analysis for cleaved PARP, a well-characterized marker of apoptosis. No cleaved PARP was detected in any of the cell lines following the single agent treatments (Figure 4A), but when dual inhibition with either ZSTK474 or PI-103 was administered, marked PARP cleavage was seen in the H3122 line but not in the other lines tested (Figure 4A).

### Effect of dual inhibition on cell signaling

The NSCLC (H3122 and H1437), breast cancer (MDA-MB231) and colon cancer (HCT116) lines, which showing major synergy upon dual inhibition, were further studied for cell signaling in response to the inhibitors. All the cell lines downregulated pAKT and its downstream target pS6 completely in response to 6h of treatment with the PI3K inhibitor ZSTK474 or PI-103 (3.3 μM) (Figure 3A). Downregulation of p4E-BP1 was also noted with all the cell lines tested, but it was complete only in the H3122 cell line (Figure 3A). Furthermore, concurrent activation of pERK1/2 was recognized in the H3122, MDA-MB231 and HCT116 cell lines during PI3K inhibitor treatment (Figure 3A). When the cell lines were treated with the MEK inhibitor CI-1040 (1 μM, 6 h), complete (H3122, H1437) or marked (MDA-MB231, HCT116) downregulation of pERK1/2 was seen (Figure 3A). This was accompanied by upregulation of pAKT in the H3122 and MDA-MB231 lines, but not by upregulation of pS6 or p4E-BP1 (H3122) (Figure 3A). p4E-BP1 was markedly upregulated in the MDA-MB231 line in response to CI-1040 treatment (Figure 3A).

When the PI3K and MEK inhibitors were administered simultaneously the inhibition of the targets was similar to that seen with single inhibitor treatment (Figure 3A). Dual inhibition was able to overcome the single inhibitor-induced stimulation of parallel pathway activation (Figure 3A). We were not able to detect any significant difference in the activity of either pS6 or p4E-BP1 following dual inhibitor treatment as compared with the single PI3K inhibitor treatments (Figure 3A).

**Figure 5:**
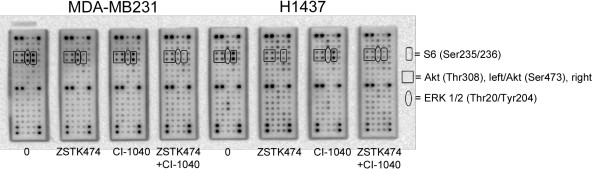
**PathScan analysis of dual inhibition of the central RTKs and signaling nodes.** The dual inhibition-sensitive MDA-MB231 and H1437 cell lines were left untreated (0), treated with 3.3μM ZSTK474, 1 μM CI-1040, or their combination for 24 h and analyzed with the PathScan antibody array assay for the phosphorylation status of 28 central RTKs (bottom 13 rows) and 11 signaling nodes (top five rows). The AKT, S6 and ERK1/2 signals are circled. The whole array target map is available through the manufacturer’s homepage (http://www.cellsignal.com/products/7982.html).

Further analysis of the dual inhibition of the central RTKs and signaling nodes was carried out with the PathScan Antibody Array, which investigates the phosphorylation status of 28 RTKs and 11 signaling nodes concurrently. Attention was focused on the dual inhibition-sensitive H1437 and MDA-MB231 lines. A low level of RTK activation was noted in untreated cells of both cell lines, H1437 showing some activity with c-MET (Figure 5), while in the signaling nodes, pAKT, S6 and ERK1/2 showed activity in both cell lines and Src activity was also noted in H1437. In the drug-treated cells, ZSTK474 (24 h) was able to inhibit both AKT and S6 phosphorylation, S6 showing a more pronounced effect (Figure 5). Furthermore, ZSTK474 induced a marked broad feedback RTK activation in the H1437 cell line (Figure 5). CI-1040 (24 h) effects were limited to the inhibition of ERK1/2 activity. When dual inhibition with ZSTK474 and CI-1040 was administered, downregulation of both pAKT/S6 and ERK1/2 was noted, but otherwise no marked difference was evident relative to the single agent treatments (Figure 5). The results suggest specificity of the inhibitors for their targets and the existence of broad feedback activation.

### Alternative dosing of dual inhibition

**Figure 6:**
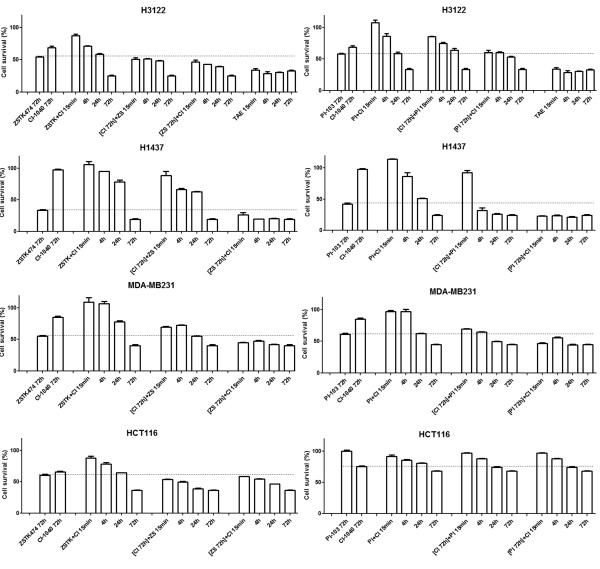
**Alternative dosing schedules for concurrent PI3K and MEK inhibition in dual inhibition**-**sensitive cancer cell lines. **The cell lines were exposed to the inhibitors (ZSTK474 and PI-103 3.3 μM, CI-1040 1 μM, or their combinations) for the times indicated and analyzed with the MTS assay after 72 h. H3122, an *ALK* translocated and ALK inhibition-sensitive NSCLC line, was also exposed to the 0.1 μM ALK inhibitor TAE684 (TAE) for the times indicated. Error bars show SD. A dashed line is inserted at the level of the single inhibitor treatment inducing maximal growth inhibition to facilitate comparison with the dual inhibition treatments.

Even though dual inhibition of PI3K and MEK was identified as an effective form of cancer therapy based on the *in vitro* models, administration of both drugs at doses inducing major downregulation of the target for long periods of time may be too toxic in a clinical setting. We therefore set out to investigate concurrent administration of PI3K and MEK inhibitors to cell lines sensitive to dual inhibition with alternative dosing schedules. The MTS assays showed that for maximal reduction in the number of living cells in all the lines, dual inhibition needed to be administered for longer periods of time. The therapy was significantly more effective when it was administered throughout the 72 h experiment as compared with 15 min, 4 h or 24 h periods (Figure 6). Interestingly, maximal cytotoxicity was seen in the *ALK* translocated H3122 line even with short courses of ALK inhibition (15 min), while similar cytotoxicity was seen with 72 h inhibition of PI3K and MEK concurrently (Figure 6), even though both approaches induced major inhibition of phosphorylated AKT and ERK in Western blots after 6 h treatments (Figure 3A).

Since the results showed that dual inhibition needed to be administered for longer periods of time for maximal cytotoxicity, we turned next to investigating whether both inhibitors are required throughout the period of exposure. The dual inhibition-sensitive cell lines were exposed to one inhibitor throughout the treatment period (72 h) while the other inhibitor was administered concurrently for 15 min, 4 h or 24 h at the beginning of the drug exposure. The results varied significantly between the cell lines tested. In the H1437 and MDA-MB231 lines concurrent inhibition of PI3K and MEK for 15 min with continued PI3K inhibition for 72 h achieved similar cytotoxicity to concurrent inhibition for 72 h (Figure 6). Conversely, when these lines were exposed to the MEK inhibitor throughout the treatment period, short concurrent exposures (15 min, 4 h or 24 h) to PI3K inhibitors did not induce any comparable cytotoxicity (Figure 6). On the other hand, the effects of dual inhibition with PI-103 occurred faster in the H1437 line than with ZSTK474, since shorter exposures to the drug (24 h) seemed to be sufficient for maximal cytotoxicity as compared with 72h of ZSTK474 (Figure 6). In the case of the H3122 and HCT116 lines, both the PI3K and MEK inhibitors needed to be administered throughout the treatment period for maximal cytotoxicity (Figure 6).

We next investigated alternative dosing of the dual inhibition of cell signaling. The dual inhibition-sensitive lines were exposed to the PI3K inhibitors and MEK inhibitor concurrently for 15 min, after which treatment was continued with a single inhibitor for the remainder of the 6 h period. pAKT downregulation was complete or nearly complete when the cells were treated for only 15 min and with PI3K inhibitors for 6 h (Figure 3B), while conversely, pERK1/2 recovered completely in 6 h when the cells were treated with the MEK inhibitor for 15 min (Figure 3B). Interestingly, we were able to see some recovery in the activity of the downstream targets of AKT when the PI3K inhibitors were administered for 15min despite the remaining pAKT downregulation. The pS6 signal was able to recovery in the MDA-MB231 (with ZSTK474) and HCT116 (with both PI3K inhibitors) lines after short PI3K administration (Figure 3B). Furthermore, p4E-BP1 recovery was noted in the H3122 (with ZSTK474), MDA-MB231 (with ZSTK474), and HCT116 (with both PI3K inhibitors) lines (Figure 3B). Interestingly, MEK inhibitor treatment induced upregulation of p4E-BP1 in the MDA-MB231 line (Figure 3A), and marked downregulation p4E-BP1 was noted only with PI-103 (PI3K and mTOR inhibitor) in the alternative dosing experiments, but not with ZSTK474 (with a PI3K inhibitor alone) (Figure 5), suggesting mTOR-mediated activation of 4E-BP1 in response to MEK inhibition. TAE684, an ALK inhibitor, treatment was also included in the experiments conducted with the H3122 line, and this induced comparable pAKT, pERK1/2, and pS6 downregulation to that achieved with dual inhibition, whereas no change in p4E-BPI was noted (Figure 3B). Some recovery of pAKT and pS6 was seen after a short treatment with TAE684 (Figure 3B).

We went on further to analyze whether the alternative dosing could also result in apoptosis in the H3122 cell line, the only line identified as inducing apoptosis in response to dual inhibition. When the cells was treated for 15 min with dual inhibition and treatment with either the PI3K inhibitors or the MEK inhibitor was continued for 48 h, marked PARP cleavage was seen in all the treatments (Figure 4B). Furthermore, 15 min treatment with an ALK inhibitor resulted in marked PARP cleavage (Figure 4B). Cleaved PARP results were further verified with western blot analysis for cleaved caspase-3, another marker for apoptosis. Cleaved caspase-3 was detected with concurrent PI3K and MEK, or ALK inhibition while no signal was seen in PI3K or MEK inhibitor treatments. Conversely to cleaved PARP, the cleaved caspase-3 signal was much lower in alternative dosing schedules compared to continuous, concurrent PI3K and MEK inhibition (Figure 4C).

## Discussion

The PI3K-AKT and RAS-RAF-MEK-ERK signaling pathways are thought to be the central mediators of oncogenic signals in solid malignancies. Multiple inhibitors targeting PI3K, AKT, RAF and MEK are under development for cancer therapy, but early-phase clinical trials suggest that the single agent efficiency of such inhibitors seems to be limited, except in the case of the *Raf* mutant melanoma, where both RAF and MEK inhibitors have high clinical activity. There is preclinical evidence that combining the inhibitors of both pathways provides more efficient cancer therapy [2,4,6,7], and some early-phase clinical trials are under way to test this approach.

We investigated here the dual pharmacological inhibition of PI3K and MEK in NSCLC cell line models with specific oncogenic genotypes. All the cell lines tested were highly responsive to single-agent PI3K inhibitors, showing a strong correlation with maximal target inhibition. This suggests that the PI3K-AKT pathway has a central role in transmitting oncogenic signals from various upstream sources, and therefore the responses to pathway inhibition are not limited to any specific cancer genotype. Furthermore, the data suggest a central role for pathway activation in the proliferation of carcinomas. The cytotoxicity of PI3K inhibitors seemed to be comparable when a PI3K (ZSTK474) or PI3K/mTOR (PI-103) inhibitors alone were used, suggesting that only PI3K inhibition matters for cytotoxicity, as administration of the MEK inhibitor seemed to have limited activity or none at all in the models tested. Two out of the twelve cell lines tested showed significantly increased cytotoxicity in response to the concurrent administration of PI3K and MEK inhibitors. Analogously to previous studies, the activity of dual inhibition was not associated with any specific oncogenic genotype, since ALK translocation-positive (H3122) and triple-negative (H1437) cell lines were the most responsive ones [6]. In MEK inhibition-sensitive models. such as triple-negative breast or *KRas* mutant colorectal cancers have shown additive cytotoxicity or reversal of resistance when MEK inhibitors have been combined with inhibitors of the PI3K-AKT-mTOR pathway [4,7]. It is interesting to note that the dual inhibition-sensitive NSCLC lines identified here showed some cytotoxicity in response to low concentrations of MEK inhibitors (<1 μM), thereby differing from the other lines tested, which showed no response or a response only to high concentrations of the inhibitor. Furthermore, the *KRas*, *EGFR* and *ALK* wild-type cell H1437 is of a rare oncogenic genotype, a *MEK1* mutant, and has previously been identified as being sensitive to MEK inhibitor treatment alone [21]. Based on the current data and previously reported findings, one could speculate that dual PI3K and MEK inhibition therapy could be the most efficient for cancers that show some dependence on MEK signaling for their proliferation or survival. Mechanistically, sensitivity to dual PI3K and MEK inhibition remains to be elucidated. It is likely that the responses are not associated with any specific oncogenic genotype but rather with inhibition of the effects of feedback activation induced by the inhibition of one pathway on the other. If this also holds good *in vivo*, it is likely to make the selection of patients for such treatment difficult, since no predictive biomarkers of feedback activation exist.

Even though dual inhibition of PI3K-AKT and MEK has been identified as an effective cancer therapy in preclinical models, it questionable whether this therapy is tolerable in a clinical setting concentrations high enough to achieve sufficient target inhibition. Early-phase clinical trials are in progress to test different doses and dosing schedules, but the optimal administration for maximal efficiency and tolerability remains to be elucidated. In the light of recent data from the ASCO 2012 Annual Meeting, PI3K and MEK inhibitor combination treatments are now being tested in concurrent and intermittent schedules [22,23]. The tolerability of intermittent administration may enable higher doses of the agents to be administered than with continuous concurrent treatment [23]. The cell line model data presented here suggest that even short courses of concurrent administration can cause marked cytotoxicity and/or apoptosis. Two out of the four dual inhibition-sensitive cell lines showed comparable cytotoxicity to that achieved with continuous administration of dual inhibition when the MEK inhibitor was administered for short periods (15 min) in combination with continuous PI3K inhibitor treatment. The increased cytotoxicity occurred even though the effects of the MEK inhibitor were quickly reversed (<6 h) after wash-out of the drug. Meanwhile H3122, an *ALK* translocated cell line, showed apoptosis in response to short concurrent administration of the drugs even though longer concurrent administration led to maximal cytotoxicity. Interestingly, short courses of ALK inhibition (15 min) induced comparable cytotoxicity to long administration of either an ALK inhibitor or a dual inhibitor combination, even though the ALK inhibitor is reversible in its mode of action and some recovery of the target inhibition is known to occur within 6h. In the light of our *in vitro* data, one could hypothesize that even a short course of dual inhibitor administration could have similar clinical effects with better tolerability. Analogously, a recent work has shown that intermittent administration of concurrent PI3K and MEK inhibition can induce robust growth inhibition in cancer cell lines [24]. Better alternative dosing schedules for achieving clinical tolerability could also enable the use of higher doses of the drugs, leading to stronger inhibition of the target. Short but more significant target inhibition is likely to be more efficient than sub-maximal inhibition for longer periods. Our data point to the importance of maximal inhibition of the target and a preferential role for longer PI3K-AKT pathway inhibition when dual inhibition is used. These data are based only on *in vitro* models, however, and correlation with the *in vivo* situation is not always a straightforward matter.

The interconnectivity of the PI3K-AKT-mTOR and RAS-RAF-MEK-ERK pathways makes the idea of their concurrent dual inhibition an appealing one. The present cell signaling experiments also showed high interconnectivity of these two pathways, since in many instances inhibition of one pathway resulted in concurrent feedback activation of the other. Furthermore, another MEK inhibition-induced feedback mechanism was identified in the MDA-MB231 cell line which led to the activation of 4E-BP1 independently of PI3K-AKT. Previous studies have suggested that the PI3K-AKT-mTOR and RAS-RAF-MEK-ERK pathway signals converge at 4E-BP1, and that its inhibition may be a major determinant of the efficiency of dual inhibition [25]. Conversely, we did not find any correlation between the efficiency of dual inhibition and 4E-BP1 downregulation, since the 4E-BP1 signal correlated significantly only with PI3K-AKT-mTOR activity and cytotoxicity occurred without it being downregulated. In also, some of the treatment schedules induced marked cytotoxicity in the H3122 and MDA-MB231 cell lines without the induction of any marked 4E-BP1downregulation.

## Conclusions

The most important findings to emerge from this investigation of the concurrent dual inhibition of PI3K and MEK for cancer therapy purposes are the fact that alternative dosing schedules result in comparable cytotoxicity to that achieved with continuous treatment schedules, and that the responses to dual inhibition can be achieved in multiple cancer genotypes. The present preclinical data may offer new leads for clinical progress towards more efficient and tolerable cancer therapies.

## Competing interests

The authors’ declare no competing interests.

## Authors’ contributions

JPK conceived the study design and was coordinated the work. JPK and EJ carried out the laboratory experiments, participated in the gathering, analysis and interpretation of the data, and drafted, read and approved the final version of the manuscript. All authors read and approved the final manuscript.

## Pre-publication history

The pre-publication history for this paper can be accessed here:

http://www.biomedcentral.com/1471-2407/12/612/prepub
